# The quantitative impact of metabolism‐inhibiting drugs on the occurrence of adverse drug reactions—A backward selection approach

**DOI:** 10.1002/bcp.70494

**Published:** 2026-02-20

**Authors:** Judith Berres, Justyna Wozniak, Hannah Fölsch, Anja Knueppel‐Ruppert, Verena Graeff, Andrea Kriegisch‐Stumpf, Harald Dormann, Julia C. Stingl, Katja S. Just

**Affiliations:** ^1^ Institute of Clinical Pharmacology University Hospital RWTH Aachen Aachen Germany; ^2^ Central Emergency Department Hospital Fürth Fürth Germany; ^3^ Central Emergency Department University Hospital Augsburg Augsburg Germany; ^4^ Department of Interprofessional Hospital Development University Hospital Augsburg Augsburg Germany; ^5^ Central Emergency Department Hospital Ingolstadt Ingolstadt Germany; ^6^ Department of Clinical Pharmacology and Pharmacoepidemiology Heidelberg University Hospital Heidelberg Germany

**Keywords:** adverse drug reaction, drug inhibition, falls, haemorrhages

## Abstract

**Aim:**

The quantitative effect of several inhibitory drugs on the development of adverse drug reactions (ADRs) is currently difficult to estimate. Our aim was to identify metabolic pathways, which, when inhibited, increase the risk for certain ADRs, and to use this system to consider comedication at individual level.

**Methods:**

Data of a prospective multicentre cohort of ADRs on emergency department admissions were used (ADRED‐study, trial registration: DRKS00008979). A score system to rate the burden of inhibition on pathways was implemented to calculate individual burdens per patient. These cumulative burden scores were used as predictors in binary logistic regressions to identify pathways, which were significantly positively correlated with the ADRs falls and haemorrhages, respectively.

**Results:**

Regarding falls, CYP2D6, OCT2 and carboxylesterase 1 were identified as relevant metabolic pathways with cumulative inhibitory burden. Concerning haemorrhages, CYP2C19, CYP3A4, MRP4, OAT2, OATP1B1 and carboxylesterase 1 were determined. Within relevant pathways for the occurrence of falls, common fall‐risk‐increasing drugs (FRIDs) and associated drugs were found, as well as bleeding inducing drugs within relevant pathways for the occurrence of haemorrhages.

**Conclusion:**

Our approach identifies plausible inhibited pathways, raising awareness that a high burden of pathway inhibition can contribute to the occurrence of ADRs. Further validation in a second dataset is necessary.

**What is already known about this subject?**

- Drug–drug interactions increase the risk of adverse drug reactions (ADRs).
- Inhibition of a degradation pathway can provoke substrates of this pathway to cause ADRs due to pharmacokinetic interactions.
- Comedication varies widely between patients and is difficult to be captured with a single fixed metric.

**What this study adds?**

- CYP2D6, OCT2 and carboxylesterase 1 were identified as relevant metabolic pathways with cumulative inhibitory burden in falls.
- CYP2C19, CYP3A, MRP4, OAT2, OATP1B1 and carboxylesterase 1 were determined as relevant pathways with inhibitory burden in haemorrhages.
- Cumulative burden scores were developed as new quantitative measure of comedication.

## 1 INTRODUCTION

In 2008, the European Commission estimated that 197 000 deaths per year in the EU are caused by adverse drug reactions (ADRs), causing costs of 79 billion €.^1^ Furthermore, ADRs result in a not to be neglected quantity of hospitalized patients; 5–8.5% of emergency department admissions can be attributed to ADRs.^2^, ^3^, ^4^

A high proportion of ADRs is considered as avoidable^3^—patients' harm as well as high economic costs could be prevented. The IATROSTAT‐ECO study estimated the mean total cost per patient due to hospital administration because of preventable ADRs at €4542 ± €2622 in French hospital patients with 2023 tariffs.^5^ Still, in clinical situations, it is barely possible to predict which patient will suffer from an ADR. Well‐known risk factors include age,^6^ frailty,^6^ female sex,^7^, ^8^ and polypharmacy.^9^

Beside problems with application and dosages, drug–drug interactions (DDI), either pharmacodynamic or pharmacokinetic, play a central role in the appearance of ADRs.^10^ First DDIs occur, if the drugs show either direct or indirect additive, synergistic or antagonistic effects on receptors. Second include changes in adsorption, distribution, metabolism and excretion of a drug.^11^ A large number of DDIs ensue due to inhibition or induction of drug metabolizing enzymes as cytochrome P450 (CYP) enzymes^12^ and drug transporters.^13^ Consequently, a higher number of medications in a patient are associated with increased risk of ADRs, as shown for instance in nursing home residents^14^ and emergency department patients.^15^

In multi‐medicated cases, today's Clinical Decision Support Systems consider only the interactions between two of the taken drugs separately as ‘pairs’, in other words drug A + B, A + C and B + C, but not the whole combination A + B + C at once. This leads to overestimation or underestimation of the combined pharmacokinetic impact of several inhibitors or inducers on a pathway. Subsequently, no overall picture is obtained of which combinations could be responsible for clinically relevant changes of drug exposure, causing ADRs. Instead, over‐alerting is generated, which may entail alert fatigue, resulting in clinicians overriding or ignoring alerts.^16^ Whereas changes in plasma levels can be calculated using physiologically‐based pharmacokinetic models (PBPK models), and inhibitory influence on enzyme activity by use of Michaelis–Menten equations, the use of a clinical important outcome such as ADRs could improve the clinical relevance and implementation into clinics.

The question of how likely the inhibition of a metabolic pathway could cause an ADR is highly relevant and prominent in clinician's everyday life. Especially when new drugs are added to an existing drug regimen or if multiple drugs are newly prescribed simultaneously, a quickly executed evaluation of the pathway‐depending burden would be a help to better assess the risk of ADRs caused by DDIs.

In case of additive pharmacodynamic DDIs, some approaches, such as for the anticholinergic burden, exist. Based on clinical empirical knowledge, the impact of drugs on the anticholinergic system is represented by single scores of 0–3, which are added to a patient‐individual burden. A high anticholinergic burden can lead to dry mouth, constipation and urinary retention but also neuropsychiatric effects, cognitive impairment, increased falls and more frequent hospitalization.^17^, ^18^, ^19^ Although seven different expert‐based anticholinergic rating scales exist but no standardized tool for measuring,^20^ the anticholinergic burden is a common system to evaluate the risk of anticholinergic mediated ADRs. A comparable approach to estimate the clinical impact of the pharmacokinetic burden on drug pathways to the best of our knowledge is lacking. To address ADRs caused by pharmacokinetic interactions, two key aspects need to be considered: First, it is essential to identify which drugs inhibit specific metabolic pathways in a patient and to what extent. Second, it is important to identify which drugs act as substrates on these pathways and are consequently prone to change plasma levels and affect the likelihood of an ADR.

The aim of this study was to detect relevant metabolic pathways for the occurrence of ADRs and to investigate the impact of the burden of inhibition on relevant pathways.

## 2 METHODS

### 2.1 Study population and dataset

Data of the ADRED‐study were used. In the multi‐centric prospective observational trial ADRED (Adverse Drug Reactions in Emergency Departments, trial registration: DRKS00008979), patients were collected in six German emergency departments of tertiary care hospitals.^21^ Patient cases were included with an age equal or older than 18 years, presenting with symptoms recognized as ADR according to the standardized WHO‐UMC system by trained study staff.^2^, ^21^ Patients provided written informed consent. Ethical approval was granted by the ethical committee of the University of Bonn (202/15).

Further information can be found elsewhere.^2^, ^21^

The dataset included information on basic patient characteristics, ICD‐10 diagnoses at time of admission and discharge, lab values and information on the current drug treatment with ATC‐codes and WHO‐UMC causality assessment at time of admission, as well as ADR symptoms at admission coded according to MedDRA version 22.0.

### 2.2 Data curation

The ADRED cohort (*n* = 7967) was first reduced by all patients who were unable to identify all drugs with brand names or active pharmaceutical ingredients, recognizing only groups as, for example, analgetic or oral contraceptive. In these patients, the exact affected pathways were unclear (*n* = 242).

In the remaining medication data, all medication—but not inevitably patients—with non‐systemic drugs, phytopharmaceuticals, nutritional supplements, vitamins, trace elements, homoeopathic and anthroposophical remedies was excluded, because no systemic effect was expected (*n* = 291). Finally, all patients with omitted medication due to ADRs or due to noncompliance were excluded, whereby the first study cohort was excluded completely due to no systematic capture of omissions, resulting in a cohort of *n* = 4581 cases for the following analysis.

### 2.3 Assessment of metabolic pathways and modulating effects

**FIGURE 1:**
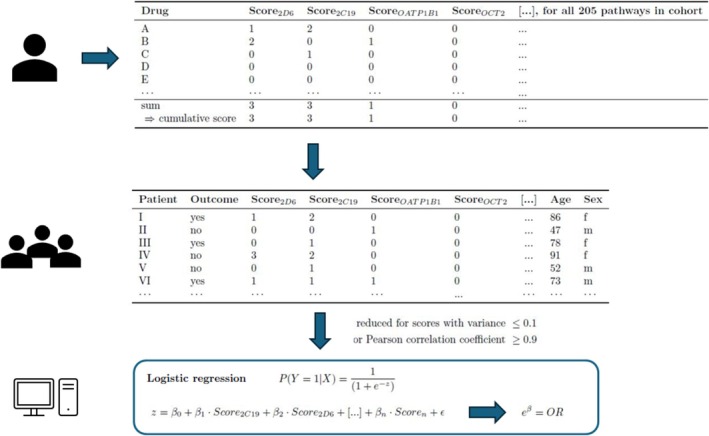
Flowchart displaying calculation of the cumulative scores, composition of dataset and logistic regression.

To assess the metabolism and interaction data of each drug documented in the ADRED study, the mediQ database, a Swiss database containing information on drugs and DDIs, was used.^22^ In total, we identified *n* = 205 pathways as relevant for the drugs documented in the ADRED database by the use of the mediQ software. To determine a measure for the inhibition strength per patient and pathway, a score system was implemented: mediQ offers a rating of the inhibitor strength with values from 0/3 to 3/3, with 0/3 indicating inhibition without clinical relevance, 1/3 weak inhibition, 2/3 moderate inhibition and 3/3 strong inhibition. The mediQ information on the modulating effect of each drug taken was used to calculate first a single score for each pathway the drug is inhibiting. Thereby, a weak inhibition was assigned a score of 1, a moderate was assigned a score of 2 and a strong inhibition was assigned a score of 3. Inhibitions without clinical relevance (0/3) were not considered relevant in this step. Subsequently, the single scores per patient were added to a cumulative pathway score for each patient; see Figure 1.

### 2.4 Assessment of symptoms as fall or haemorrhage

We chose two clinical outcomes due to their frequent occurrence and clear endpoint in the dataset, namely, falls documented as preferred term (PT) using MedDRA (Code 1001673) and bleeding according to the standardized MedDRA Query ‘haemorrhage terms (excluding laboratory terms)’ (Code 20000039), Version 26.1. An insufficient number of patients had available laboratory values to allow application of the broad version of the MedDRA Query for haemorrhage.

### 2.5 Classification of drugs into fall‐ and bleeding‐inducing agents

To determine the impact of drugs on the genesis of falls as well as haemorrhages, given drugs were classified according to their known ADRs as fall‐inducing or bleeding‐inducing. Fall‐risk increasing drugs were defined according to the STOPPFall criteria of the European Geriatric Medicine Society (EUGMS) and Finish group on Fall‐Risk‐Increasing drugs and systematic reviews.^23^, ^24^, ^25^, ^26^ Drugs of interest regarding bleeding as ADRs were defined as antithrombotic (ATC B01), non‐steroidal anti‐inflammatory drugs (ATC M01A) and selective serotonin‐reuptake inhibitors (ATC N06AB).

### 2.6 Data analysis and statistics

#### 2.6.1 Descriptive analysis

Descriptive analysis of population characteristics was conducted. The Kolmogorov–Smirnoff test was used to determine normal distribution in continuous variables. As all continuous variables were not normally distributed, they are shown in median and 25th/75th percentile. Categorical variables are shown in absolute numbers and percentages.

#### 2.6.2 Modelling falls and haemorrhages as logistic regressions

For both outcomes, fall and haemorrhage, a binary logistic regression with backward elimination was performed. The dataset contained 205 pathways overall, which were assigned a cumulative score due to inhibition in at least one patient case. Yet, complemented with age and sex as further predictors, the data showed too high multicollinearity to undergo a binary logistic regression. Hence, the 205 cumulative scores were reduced first for all cumulative scores with a variance ≤0.1—by this, predictors with a very low number of nonzero cumulative scores were removed. This reduced the cumulative scores to 34 different pathways. The remaining predictors were tested for binary correlation using the Pearson correlation coefficient. For all correlated columns (Pearson ≥ 0.9), one was removed to avoid redundancy. This resulted in a set of 31 cumulative pathway scores plus age and sex to be used as predictors in following models.

With this dataset, a backward stepwise selection was performed, using the backward elimination (likelihood ratio) method in SPSS to identify relevant pathways that are inhibited shown by cumulative burden and associated with the ADRs fall and haemorrhage, respectively. The backward elimination method bases the removal testing on the probability of the likelihood‐ratio statistic. Results are given as odds ratios (OR) and 95% confidence intervals (CI). For details on mathematical interpretation see Supporting Information S1.

Data analysis and statistics were performed using Python 3 via Jupyter Lab Version 3.6.5 and IBM SPSS Statistics, Version 29.0.0.0 (IBM Corp., Armonk, NY, United States). Data processing utilized libraries such as pandas (v2.1.4), numpy (v1.24.3), matplotlib (v3.7.2), seaborn (v0.12.2), scikit‐learn (v1.6.1) and statsmodels (v0.14.0).

### 2.7 Nomenclature of targets and ligands

Key protein targets and ligands in this article are hyperlinked to corresponding entries in http://www.guidetopharmacology.org and are permanently archived in the Concise Guide to PHARMACOLOGY 2021/22.^27^, ^28^

## 3 RESULTS

### 3.1 Characteristics of the cohort and drug frequency

The cohort showed a median age of 75 (62; 81) with a nearly equal sex ratio (female: 49.5%, *n* = 2268). Patients presented with a median number of symptoms of 3 (2; 5) and a median number of systemic drugs of 7 (4; 10).

The most frequently documented drugs in the study‐cohort included pantoprazole (35.5%, *n* = 1672), torsemide (31.6%, *n* = 1448) and acetylsalicylic acid (30.0%, *n* = 1375), more details can be found in Table 1.

**TABLE 1:** Characteristics of the study population (*N* = 4581) with adverse drug reactions (ADRs) leading to emergency department admissions.

Characteristic	
Age in years, median (25th; 75th percentile)	74 (62; 81)
Sex, *n* (%)	
Female	2268 (49.5)
Male	2313 (50.5)
No. of systemic drugs taken, median (25th; 75th percentile)	7 (4;10)
No. of symptoms, median (25th; 75th percentile)	3 (2;5)
15 most frequent drugs, *n* (%)	
Pantoprazole	1672 (36.5)
Torsemide	1448 (31.6)
Acetylsalicylic acid	1375 (30.0)
Ramipril	1149 (25.1)
Metoprolol	1083 (23.6)
Metamizole sodium	1062 (23.2)
Levothyroxine	909 (19.8)
Bisoprolol	902 (19.7)
Atorvastatin	847 (18.5)
Amlodipine	753 (16.4)
Candesartan	724 (15.8)
Simvastatin	668 (14.6)
Allopurinol	610 (13.3)
Metformin	566 (12.4)
Hydrochlorothiazide	560 (12.2)
Apixaban	547 (11.9)

### 3.2 Evaluation of the cumulative burden scores system

**FIGURE 2:**
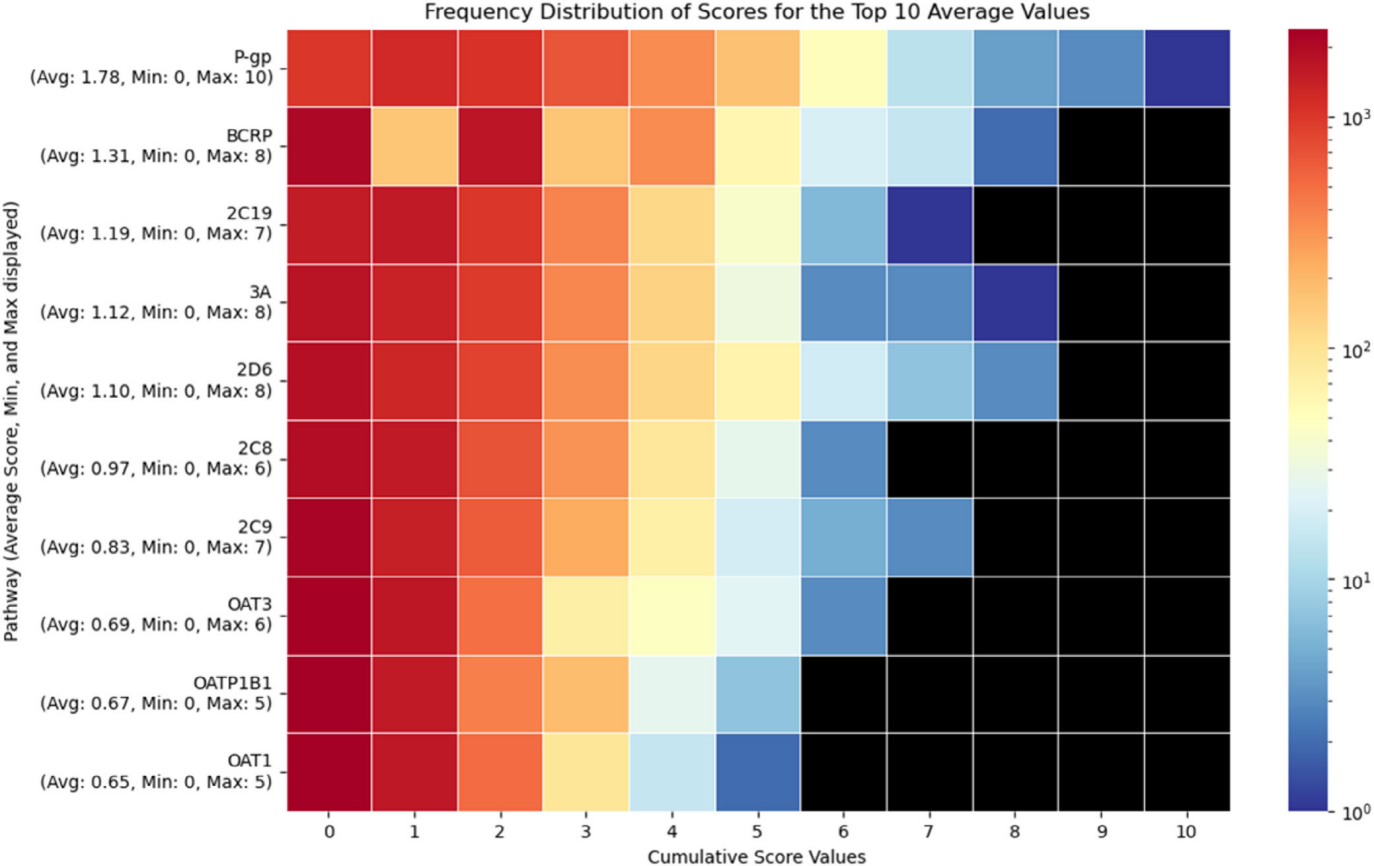
Pathways with the 10 highest average cumulative burden scores and distribution of score values. A logarithmic colour scale was used to improve contrast and highlight low‐frequency score values across the full 0–10 range. Black squares represent not existing values. The average score on the pathway, minimum and maximum score are displayed on the left‐hand side.

The cumulative burden scores of each pathway present in the data were compared regarding frequency of pathway inhibition and score value as measure for the inhibition strength. The highest score values were reached on P‐gp, BCRP, CYP3A and CYP2D6, whereas the highest average score values were prominent on P‐gp, BCRP and CYP2C19. Details can be found in Figure 2.

### 3.3 Model results and pharmacological linkage

The logistic regression with fall as outcome showed both the model itself (χ^2^ = 127.92, *p* < .001, *n* = 4581), and all coefficients were significant; see Supporting Information S2. Positive ORs display that the increasing burden is associated with a higher chance for a fall, whereas negative ORs do not display a protective effect but can be explained due to the cohort structure: Patient cases without a fall as ADR suffered other ADRs; hence, other pathways were relevant.

Table 2 shows model results for each cumulative burden score of a pathway, which was significantly positively associated with the occurrence of falls and haemorrhages as ADRs.

**TABLE 2:** Significant positively associated inhibited pathways with the occurrence of the adverse drug reactions falls and haemorrhages using logistic regression models with backward selection.

Cumulative burden score of pathways	Outcome falls, OR [95% CI]	Outcome haemorrhages, OR [95% CI]
CYP2D6	1.18 [1.06–1.30]	–
OCT2	1.39 [1.10–1.74]	–
Carboxylesterase 1	2.42 [1.18–4.95]	1.32 [1.08–1.60]
CYP2C19	–	1.24 [1.12–1.36]
CYP3A	–	1.10 [1.00–1.22]
MRP4	–	1.47 [1.17–1.84]
OAT2	–	1.50 [1.14–1.98]
OATP1B1	–	1.18 [1.08–1.29]

*Note*: Models adjusted for age (in years) and female sex. Thirty‐one cumulative pathway scores were included in models and reduced by backward selection.

Abbreviations: –, no significant positive association; CI, confidence interval; OR, odd's ratio.

For falls, the CYP2D6 pathway (OR 1.18, 95% CI 1.06–1.30), the OCT2 pathway (1.39, 1.10–1.74) and the carboxylesterase 1 were positively associated (2.42, 1.18–4.95).

Hereinafter, appearing substrates of the relevant pathways were evaluated to enable a pharmacological interpretation of the model results. Details on the substrates of CYP2D6, OCT2 and carboxylesterase 1 present in the ADRED cohort with a frequency of at least 2% are shown in Supporting Information S3. Several FRIDs occurred including antidepressants, antipsychotics, opioids and alpha‐receptor blockers.

Regarding the occurrence of haemorrhage, the logistic regression showed likewise that the model itself (χ^2^ = 395.489, *p* < .001, *n* = 4581), and all coefficients were significant; see Supporting Information S4.

Inhibition of the CYP2C19 and CYP3A‐pathways was associated with increased odds of bleeding (1.24, 1.12–1.36 and 1.10, 1.00–1.22). Furthermore, the inhibition of the transporters MRP4 (1.47, 1.17–1.84), OATP1B1 (1.18, 1.08–1.29), OAT2 (1.50, 1.14–1.98) and the inhibition of carboxylesterase 1 (1.32, 1.08–1.60) were significantly associated with bleeding ADRs (Table 2).

Once more, within the substrates of the relevant pathways were several drugs known to cause haemorrhages as side effect, including anticoagulants, SSRIs and prednisolone. Same applied for drugs commonly prescribed with an anticoagulant. Detailed information hereon is provided in Supporting Information S5.

## 4 DISCUSSION

In this study, we offer a new viewpoint to focus on the overall pharmacokinetic inhibitory pathway burden as explanation for the occurrence of ADRs regardless of the used single drugs. Only the inhibitory strength is taken into account to determine the pathway burden. With this approach, we were able to identify certain drug pathways as being relevant for the occurrence of the important ADRs falls and haemorrhages. Interestingly, different pathways were associated with comedication in falls compared to haemorrhages.

The cumulative burden scores of CYP2D6, OCT2 and carboxylesterase 1 were significantly positively associated with the occurrence of falls as ADR. Strictly speaking, an increase of the cumulative burden score of CYP2D6, for example, by adding a weak inhibitor, increased the odds to fall by 18% in our model. CYP2D6 is responsible for ~20% of commonly used drug metabolism, although it comprises only 2–4% of overall hepatic CYP quantity.^29^ Our finding is in line with knowledge on the importance of CYP2D6 for metabolism of FRIDs, with common FRIDs in our cohort such as mirtazapine, oxycodone, quetiapine, risperidone, citalopram, tramadol or escitalopram.^23^, ^24^, ^25^, ^26^, ^30^

OCT2 and carboxylesterase 1 were significant pathways identified by our model for falls that cannot be clearly linked to substrate status of FRIDs. OCT2 is an organic cation transporter located in the basolateral membrane of renal tubular cells. It is involved in eliminating a broad range of prescription drugs and known to contribute in the interindividual drug exposure and elimination variablility.^31^ Besides, it is expressed in pyramidal cells of cerebral cortex and hippocampus, taking part in monoamine neurotransmitter uptake and clearance.^31^ There is no evidence yet for a direct correlation between OCT2 inhibition and increased fall risk. It should be seen rather as an index effect, because OCT2 inhibition is frequent among common drugs, so that an increased OCT2 inhibition can be seen as a sign for polypharmacy. Kido et al.^32^ showed 244 drugs of 910 tested drugs from a prescription drug library inhibited OCT2. Furthermore, the OCT inhibition is a standard test for the drug approval at FDA and EMA.^33^, ^34^ All considered substrates of OCT2 and carboxylesterase 1 except trimethoprim, namely, metformin, tiotropium bromide, carvedilol and clopidogrel, are common substrates in polymedicated patients.^35^, ^36^, ^37^, ^38^, ^39^ Polypharmacy is well known associated with falls,^40^ yet it is questionable, if this effect arises on the higher amount of FRIDs with increasing drug number^41^ or on the higher multimorbidity of the patients with polypharmacy as an index effect.^42^ Additionally, carboxylesterase 1 is a relevant pathway in clopidogrel metabolism, and further, clopidogrel is a weak inhibitor of carboxylesterase 1. As the mortality, length of stay and complication rate are higher in geriatric fall trauma patients on clopidogrel,^43^ a possible bias might be a more frequent hospitalization rate after falls within patients under antiplatelet therapy.

Regarding haemorrhages, the cumulative burden score of OAT2 provided the highest OR. Simply spoken, an increase of the cumulative burden score of OAT2 by adding a weak inhibitor increases the odds of haemorrhage by 50%. OAT2 is an organic anion transporter, located particularly on the basolateral and apical membrane of renal proximal tubule cells and the sinusoidal membrane of hepatocytes.^44^ It is currently considered as a transporter of emerging relevance for drug therapy,^45^ transporting a wide number of endogenous and exogenous substances. Yet the role of OAT2 in clinical drug disposition and interactions is little understood, and sufficient, selective in vivo inhibitors are currently missing.^44^ Therefore, it is of high interest that OAT2 was identified as relevant pathway in our haemorrhage model.

Except for OATP1B1, all mentioned pathways contain substrates known to cause haemorrhage as side effect. OATP1B1 substrates occurring in more than 2% of the cohort included torsemide, atorvastatin, simvastatin, rosuvastatin, ezetimibe and valsartan. None of these substrates contains increased bleeding propensity as ADRs. The impact can be again explained given that all these drugs are commonly prescribed together with an anticoagulant or antiplatelet therapy.^36^

An exception of the simple interpretation applies to the prodrug clopidogrel. Clopidogrel is metabolized mainly by CYP2C19, 1A2, 2B6 and 3A4 to the first metabolite 2‐oxo‐clopidogrel, which is further metabolized by CYP2C19, 3A4 and GSH into the active metabolite. Besides, several side pathways including carboxylesterase 1 lead to inactive metabolites. The relative importance of the individual enzymes is still controversially discussed in the literature.^46^, ^47^, ^48^ Nonetheless, an inhibition of the activating pathway leads to lower plasma levels of the first and second (active) metabolite, decreasing the risk of haemorrhages as ADR. On the other hand, an inhibition on the side pathways, for example, carboxylesterase 1, causes a higher amount of clopidogrel to be metabolized via the activating pathway, possibly leading to an increase in the active metabolite and a subsequent higher risk of haemorrhages.

As an initial proof of concept, our models exhibit some limitations. We refrained from using a quantitative distinction of every inhibitor based on in vitro values, for example, *K*_*m*_ values, due to questionable transferability into clinical effects and dependence on in vitro measuring methods. Hence, our score is based on a simple, yet valid and potentially clinically relevant classification according to the mediQ database.^22^ We decided for the system of weak, moderate and strong inhibitors to focus solely on the pharmacokinetic interaction. Saturation effects of enzymes are not considered.

Our approach to convert weak, moderate and strong inhibitors into single scores of 1, 2 and 3 and the subsequent addition of all single scores to a cumulative pathway burden is carried out as—to our best knowledge—no valid quantification method for the burden on a single pathway is known. Obviously, another single score value system or a more complex function than summation is conceivable, but our simple approach shows reasonable and well interpretable results.

In the current approach, only pathway inhibition but not induction has been considered. Inhibition is the predominant mechanism affecting CYP enzymes in clinically relevant drug–drug interactions.^49^ Induction on the contrary is less frequent. Besides, induction becomes apparent more slowly and persists longer, as the synthesis and degradation of new enzymes take some time.^50^ ADRED offered no longitudinal data, so it was impossible to determine how long the patients have been under the current medication at admission.

Compared to logistic regressions optimized for prediction, both models miss a good log likelihood and *R* ^2^ (see Supporting Information S1 and S3). Our aim was not to build the best predictive regression for falls and haemorrhages but to use the backward elimination process for elucidating the impact of single pathways burden.

Predictors with negative ORs were not considered as they do not display a protective effect on the odds of the outcome but can be caused by the cohort structure of patients with other ADRs. A potential circular reasoning may arise, as our models might inadvertently include a proxy for the used drugs in the cohort by using cumulative pathway scores as a predictor and subsequently substrates of the same pathways for pharmacological interpretation, especially because some substrates show inhibiting properties on the same pathway they are metabolized by. Further validation in a second dataset, desirably with further outcomes, is necessary.

Currently, the method is not applicable in clinical practice due to missing validation and refinements. A general idea for an application could be as follows: For each metabolic pathway, one or two cutoff values could be determined, above which the burden on this metabolic pathway is so high, that an ADR can be expected with high probability. In these clinical cases, the burden on this pathway should be reduced either by reducing the number of inhibitors or by changing the substrates into drugs metabolized by a less or not inhibited enzyme to avoid ADRs. This idea is comparable to the current clinical use of the anticholinergic burden.

As mentioned above, our approach focusses solely on pharmacokinetic drug–drug interactions; pharmacodynamic interactions are not considered despite their clinical relevance, as they are based on individual drug properties. For a holistic view of an individual patient, pharmacodynamic interactions as well as organ function parameters must be taken into account.

Nevertheless, interpreting the predictors with a positive OR as a proxy for a strong influence of inhibition on these pathways on the emergence of the outcome yields results that, in a second step, can plausibly be explained by the substrates processed by these pathways and their ADRs.

This is a first approach to quantify the impact of several inhibitors on the occurrence of ADRs caused by drugs metabolized by this pathway. In current clinical practice, either only the substrates are considered, or a vague rule‐by‐thumb approach to decide if another inhibitor will cause a too high inhibition leading possibly to an ADR is used. With our approach, this is shifted to a concept identifying the objective impact of inhibitors to substrate elimination and subsequent development of ADR.

In conclusion, our approach shows a convincing representation of the inhibited pathways, raising awareness that a high burden of pathway inhibition can contribute to the occurrence of ADRs. This approach may be seen as first idea for a new system of interaction analyses, which allow to include the whole comedication.

## AUTHOR CONTRIBUTIONS

J.B., J.W., H.F., A. K‐R., V.G., A.K‐S., H.D., J.C.S. and K.S.J. conducted the research. J.B. wrote the first draft of the manuscript. J.B. and K.S.J. designed the research.

## CONFLICT OF INTEREST STATEMENT

The authors declare no conflict of interest.

## Supporting information


**Data S1:** Mathematical background to interpret the beta‐coefficients in binary logistic regression and review their impact on the occurrence of falls and haemorrhages.
Data S2: Details on the model of falls.
Data S3: Frequently occurring substrates on pathways positively associated with the occurrence of falls, classification into FRID, degree of pathway contribution on substrate metabolism, and inhibitory effect.
Data S4: Details on the model of haemorrhages.
Data S5: Frequently occurring substrates on pathways positively associated with the occurrence of haemorrhages, classification into FRID, degree of pathway contribution on substrate metabolism, and inhibitory effect.

## Data Availability

The datasets of this study are available on reasonable request from the corresponding author.

## References

[1] European Commission , Strengthening pharmacovigilance to reduce adverse effects of medicines 08.05, 2025. https://ec.europa.eu/commission/presscorner/detail/de/memo_08_782

[2] Schurig AM , Bohme M , Just KS , et al. Adverse drug reactions (ADR) and emergencies. Dtsch Arztebl Int. 2018;115(15):251‐258. doi:10.3238/arztebl.2018.0251 29735005 PMC5949373

[3] Pirmohamed M , James S , Meakin S , et al. Adverse drug reactions as cause of admission to hospital: prospective analysis of 18 820 patients. BMJ. 2004;329(7456):15‐19. doi:10.1136/bmj.329.7456.15 15231615 PMC443443

[4] Laroche ML , Gautier S , Polard E , et al. Incidence and preventability of hospital admissions for adverse drug reactions in France: a prospective observational study (IATROSTAT). Br J Clin Pharmacol. 2023;89(1):390‐400. doi:10.1111/bcp.15510 36002314 PMC10087906

[5] Laroche ML , Tarbouriech N , Jai T , Valnet‐Rabier MB , Nerich V . Economic burden of hospital admissions for adverse drug reactions in France: the IATROSTAT‐ECO study. Br J Clin Pharmacol. 2025;91(2):439‐450. doi:10.1111/bcp.16266 39363642 PMC11773093

[6] Zazzara MB , Palmer K , Vetrano DL , Carfi A , Onder G . Adverse drug reactions in older adults: a narrative review of the literature. Eur Geriatr Med. 2021;12(3):463‐473. doi:10.1007/s41999-021-00481-9 33738772 PMC8149349

[7] Franconi F , Brunelleschi S , Steardo L , Cuomo V . Gender differences in drug responses. Pharmacol Res. 2007;55(2):81‐95. doi:10.1016/j.phrs.2006.11.001 17129734

[8] Tran C , Knowles SR , Liu BA , Shear NH . Gender differences in adverse drug reactions. J Clin Pharmacol. 1998;38(11):1003‐1009. doi:10.1177/009127009803801103 9824780

[9] Davies EA , O'Mahony MS . Adverse drug reactions in special populations—the elderly. Br J Clin Pharmacol. 2015;80(4):796‐807. doi:10.1111/bcp.12596 25619317 PMC4594722

[10] Maher RL , Hanlon J , Hajjar ER . Clinical consequences of polypharmacy in elderly. Expert Opin Drug Saf. 2014;13(1):57‐65. doi:10.1517/14740338.2013.827660 24073682 PMC3864987

[11] Prybys K , Melville K , Hanna J , Gee A , Chyka P . Polypharmacy in the elderly: clinical challenges in emergency practice—part I. Emerg Med Rep. 2002;23:145‐153.

[12] Lee J , Beers JL , Geffert RM , Jackson KD . A review of CYP‐mediated drug interactions: mechanisms and in vitro drug‐drug interaction assessment. Biomolecules. 2024;14(1):99. doi:10.3390/biom14010099 38254699 PMC10813492

[13] Gessner A , Konig J , Fromm MF . Clinical aspects of transporter‐mediated drug‐drug interactions. Clin Pharmacol Ther. 2019;105(6):1386‐1394. doi:10.1002/cpt.1360 30648735

[14] Field TS , Gurwitz JH , Avorn J , et al. Risk factors for adverse drug events among nursing home residents. Arch Intern Med. 2001;161(13):1629‐1634. doi:10.1001/archinte.161.13.1629 11434795

[15] Goldberg RM , Mabee J , Chan L , Wong S . Drug‐drug and drug‐disease interactions in the ED: analysis of a high‐risk population. Am J Emerg Med. 1996;14(5):447‐450. doi:10.1016/S0735-6757(96)90147-3 8765105

[16] Felisberto M , Lima GDS , Celuppi IC , et al. Override rate of drug‐drug interaction alerts in clinical decision support systems: a brief systematic review and meta‐analysis. Health Informatics J. 2024;30(2):14604582241263242. doi:10.1177/14604582241263242 38899788

[17] Hilmer SN , Gnjidic D . The anticholinergic burden: from research to practice. Aust Prescr. 2022;45(4):118‐120. doi:10.18773/austprescr.2022.031 36110165 PMC9427617

[18] Kouladjian O'Donnell L , Gnjidic D , Nahas R , Bell JS , Hilmer SN . Anticholinergic burden: considerations for older adults. J Pharm Pract Res. 2017;47(1):67‐77. doi:10.1002/jppr.1303

[19] Hwang S , Jun K , Ah Y‐M , Han E , Chung JE , Lee J‐Y . Impact of anticholinergic burden on emergency department visits among older adults in Korea: a national population cohort study. Arch Gerontol Geriatr. 2019;85:103912. doi:10.1016/j.archger.2019.103912 31386937

[20] Salahudeen MS , Duffull SB , Nishtala PS . Anticholinergic burden quantified by anticholinergic risk scales and adverse outcomes in older people: a systematic review. BMC Geriatr. 2015;15(1):31. doi:10.1186/s12877-015-0029-9 25879993 PMC4377853

[21] Just KS , Dormann H , Bohme M , et al. Personalising drug safety‐results from the multi‐Centre prospective observational study on adverse drug reactions in emergency departments (ADRED). Eur J Clin Pharmacol. 2020;76(3):439‐448. doi:10.1007/s00228-019-02797-9 31832731

[22] mediQ , Data from: mediQ‐Interaktionsprogramm, 2024; Stand: 08/2024.

[23] Seppala LJ , Wermelink AMAT , de Vries M , et al. Fall‐risk‐increasing drugs: a systematic review and meta‐analysis: II. Psychotropics. J am Med Dir Assoc. 2018;19(4):371.e11‐371.e17. doi:10.1016/j.jamda.2017.12.098 29402652

[24] Seppala LJ , van de Glind EMM , Daams JG , et al. Fall‐risk‐increasing drugs: a systematic review and meta‐analysis: III. Others. J am Med Dir Assoc. 2018;19(4):372.e1‐372.e8. doi:10.1016/j.jamda.2017.12.099 29402646

[25] de Vries M , Seppala LJ , Daams JG , van de Glind EMM , Masud T , van der Velde N . Fall‐risk‐increasing drugs: a systematic review and meta‐analysis: I. Cardiovascular drugs. J am Med Dir Assoc. 2018;19(4):371.e1‐371.e9. doi:10.1016/j.jamda.2017.12.013 29396189

[26] Seppala LJ , Petrovic M , Ryg J , et al. STOPPFall (screening tool of older persons prescriptions in older adults with high fall risk): a Delphi study by the EuGMS task and finish group on fall‐risk‐increasing drugs. Age Ageing. 2020;50(4):1189‐1199. doi:10.1093/ageing/afaa249 PMC8244563 33349863

[27] Alexander SP , Fabbro D , Kelly E , et al. The concise guide to pharmacology 2021/22: enzymes. Br J Pharmacol. 2021;178(Suppl 1):S313‐S411. doi:10.1111/bph.15542 34529828

[28] Alexander SP , Kelly E , Mathie A , et al. The concise guide to pharmacology 2021/22: transporters. Br J Pharmacol. 2021;178(1l 1(S1)):S412‐S513. doi:10.1111/bph.15543 34529826

[29] Taylor C , Crosby I , Yip V , Maguire P , Pirmohamed M , Turner RM . A review of the important role of CYP2D6 in pharmacogenomics. Genes (Basel). 2020;11(11):1295. doi:10.3390/genes11111295 33143137 PMC7692531

[30] Flockhart DA , Thacker D , McDonald C , Desta Z . The flockhart cytochrome P450 drug‐drug interaction table, accessed July 21, 2025, https://drug-interactions.medicine.iu.edu

[31] Ailabouni A , Prasad B . Organic cation transporters 2: structure, regulation, functions, and clinical implications. Drug Metab Dispos. 2025;53(3):100044. doi:10.1016/j.dmd.2025.100044 40020559

[32] Kido Y , Matsson P , Giacomini KM . Profiling of a prescription drug library for potential renal drug‐drug interactions mediated by the organic cation transporter 2. J Med Chem. 2011;54(13):4548‐4558. doi:10.1021/jm2001629 21599003 PMC3257218

[33] M12 Drug Interaction Studies , Guidance for industry, 2024.

[34] Guideline on the investigation of drug interactions, 2012.

[35] McEvoy JW , McCarthy CP , Bruno RM , et al. 2024 ESC guidelines for the management of elevated blood pressure and hypertension: developed by the task force on the management of elevated blood pressure and hypertension of the European Society of Cardiology (ESC) and endorsed by the European Society of Endocrinology (ESE) and the European stroke organisation (ESO). Eur Heart J. 2024;45(38):3912‐4018. doi:10.1093/eurheartj/ehae178 39210715

[36] Vrints C , Andreotti F , Koskinas KC , et al. 2024 ESC guidelines for the management of chronic coronary syndromes: developed by the task force for the management of chronic coronary syndromes of the European Society of Cardiology (ESC) endorsed by the European Association for Cardio‐Thoracic Surgery (EACTS). Eur Heart J. 2024;45(36):3415‐3537. doi:10.1093/eurheartj/ehae177 39210710

[37] Cosentino F , Grant PJ , Aboyans V , et al. 2019 ESC guidelines on diabetes, pre‐diabetes, and cardiovascular diseases developed in collaboration with the EASD: the task force for diabetes, pre‐diabetes, and cardiovascular diseases of the European Society of Cardiology (ESC) and the European Association for the Study of diabetes (EASD). Eur Heart J. 2019;41(2):255‐323. doi:10.1093/eurheartj/ehz486 31497854

[38] Alwafi H , Naser AY , Ashoor DS , et al. Prevalence and predictors of polypharmacy and comorbidities among patients with chronic obstructive pulmonary disease: a cross‐sectional retrospective study in a tertiary hospital in Saudi Arabia. BMC Pulm Med. 2024;24(1):453. doi:10.1186/s12890-024-03274-5 39272014 PMC11401255

[39] Johansson KS , Jimenez‐Solem E , Petersen TS , Christensen MB . Increasing medication use and polypharmacy in type 2 diabetes: the Danish experience from 2000 to 2020. Diabetes Care. 2024;47(12):2120‐2127. doi:10.2337/dc24-0011 38709662

[40] Zaninotto P , Huang YT , di Gessa G , Abell J , Lassale C , Steptoe A . Polypharmacy is a risk factor for hospital admission due to a fall: evidence from the English longitudinal study of ageing. BMC Public Health. 2020;20(1):1804. doi:10.1186/s12889-020-09920-x 33243195 PMC7690163

[41] Zia A , Kamaruzzaman SB , Tan MP . The consumption of two or more fall risk‐increasing drugs rather than polypharmacy is associated with falls. Geriatr Gerontol Int. 2017;17(3):463‐470. doi:10.1111/ggi.12741 26822931

[42] Damian J , Pastor‐Barriuso R , Valderrama‐Gama E , de Pedro‐Cuesta J . Factors associated with falls among older adults living in institutions. BMC Geriatr. 2013;13(1):6. doi:10.1186/1471-2318-13-6 23320746 PMC3566955

[43] Coleman J , Baldawi M , Heidt D . The effect anticoagulation status on geriatric fall trauma patients. Am J Surg. 2016;212(6):1237‐1242. doi:10.1016/j.amjsurg.2016.09.036 27889266

[44] Zamek‐Gliszczynski MJ , Sangha V , Shen H , et al. Transporters in drug development: international transporter consortium update on emerging transporters of clinical importance. Clin Pharmacol Ther. 2022;112(3):485‐500. doi:10.1002/cpt.2644 35561119

[45] Nies AT , Schaeffeler E , Schwab M . Hepatic solute carrier transporters and drug therapy: regulation of expression and impact of genetic variation. Pharmacol Ther. 2022;238:108268. doi:10.1016/j.pharmthera.2022.108268 35995278

[46] Sangkuhl K , Klein TE , Altman RB . Clopidogrel pathway. Pharmacogenet Genomics. 2010;20(7):463‐465. doi:10.1097/FPC.0b013e3283385420 20440227 PMC3086847

[47] Whirl‐Carrillo M , Huddart R , Gong L , et al. An evidence‐based framework for evaluating pharmacogenomics knowledge for personalized medicine. Clin Pharmacol Ther. 2021;110(3):563‐572. doi:10.1002/cpt.2350 34216021 PMC8457105

[48] Whirl‐Carrillo M , McDonagh EM , Hebert JM , et al. Pharmacogenomics knowledge for personalized medicine. Clin Pharmacol Ther. 2012;92(4):414‐417. doi:10.1038/clpt.2012.96 22992668 PMC3660037

[49] Zhao M , Ma J , Li M , et al. Cytochrome P450 enzymes and drug metabolism in humans. Int J Mol Sci. 2021;22(23):12808. doi:10.3390/ijms222312808 34884615 PMC8657965

[50] Hakkola J , Hukkanen J , Turpeinen M , Pelkonen O . Inhibition and induction of CYP enzymes in humans: an update. Arch Toxicol. 2020;94(11):3671‐3722. doi:10.1007/s00204-020-02936-7 33111191 PMC7603454

