# Supplementary material for: The quantitative impact of metabolism‐inhibiting drugs on the occurrence of adverse drug reactions—A backward selection approach
Source: Br J Clin Pharmacol. 2026 Feb 20;92(7):2203–11. doi: 10.1002/bcp.70494 (PMC13304258; doi:10.1002/bcp.70494)
Supplement: Supplementary file 1 — Data S1: Mathematical background to interpret the beta‐coefficients in binary logistic regression and review their impact on the occurrence of falls and haemorrhages. Data S2: Details on the model of falls. Data S3: Frequently occurring substrates on pathways positively associated with the occurrence of falls, classification into FRID, degree of pathway contribution on substrate metabolism, and inhibitory effect. Data S4: Details on the model of haemorrhages. Data S5: Frequently occurring substrates on pathways positively associated with the occurrence of haemorrhages, classification into FRID, degree of pathway contribution on substrate metabolism, and inhibitory effect. [file BCP-92-2203-s001.docx]

# Supplement

**S1** –Mathematical background to interpret the beta-coefficients in binary logistic regression and review their impact on the occurrence of falls and haemorrhages

**S2** – Details on the model of falls

**S3** - Frequently occurring substrates on pathways positively associated with the occurrence of falls, classification into FRID, degree of pathway contribution on substrate metabolism, and inhibitory effect

**S4** – Details on the model of haemorrhages

**S5** - Frequently occurring substrates on pathways positively associated with the occurrence of haemorrhages, classification into FRID, degree of pathway contribution on substrate metabolism, and inhibitory effect

**S1 – Mathematical background to interpret the beta-coefficients in binary logistic regression and review their impact on the occurrence of falls and haemorrhages**

The core idea of binary logistic regression is to model the probability that a given outcome belongs to a particular category, i.e. yes/no, healthy/ill, and similar. For this, a logistic function, aka sigmoid function, is used.

In general, the probability of an outcome happening can be modelled as^1^:

$$P \left( Y=1 | X \right)=\ln\left( \frac{p}{1-p} \right)=\frac{1}{\left( 1+e^{-z} \right)}$$

with P (Y=1|X) as probability that the outcome will happen (e.g. a patient is ill) under the predictors X, e as Euler’s number and z as logit.

Hereby the logit is a linear regression model:

$$z= \beta_{0}+\beta_{1}x_{1} +\beta_{2}x_{2}+\left( \ldots\right)+{\beta_{n}x}_{n}$$

with $x$ as independent variables and $\beta$ as regression coefficients.

Be aware that regression coefficients in logistic regression cannot be interpreted as in linear regression, since the connection between regression coefficient and P is not linear. As shown, the $\beta$ -coefficients are proportional to the $\ln(\frac{p}{1-p})$ of the outcome. Although this is mathematically valid, a direct clinical interpretation is lacking here.

Instead, Odds and Odds Ratios are used to interpret this connection: Odds display the ratio of the probability that the event will happen to the probability it will not happen. Odds Ratios (OR) are the ratio of two odds and display the change in relative probability of Y=1, if all variables except the belonging x are held constant. Thereby, the OR shows, which effect the increase of the independent variable $x$ for one unit has on the odd of the event Y, if all other variables are held constant. Odds ratios can be calculated from the regression coefficients in binary logistic regression:

$$OR={\frac{Odds after increasing x for one unit}{Odds before increasing x for one unit}= e}^{\beta}$$

An OR = 1 can be interpreted so that a one-unit increase of x has no impact on the probability of Y, an OR > 1 shows the increase of relative probability and an OR <1 shows a decrease.

Percentual interpretation of OR is always connected with a chance in the odds of an event, not with a change in the probability. For example, an OR = 1.2 is equivalent to a 20% increase on the odds of an event Y, if the corresponding independent variable x is increased for one unit and if all other variables are held constant.

For our models, this generalized equation was adapted as following:

$$z=\beta_{0}+\beta_{1}Score_{A}+\beta_{2}Score_{B}+\left( \ldots\right)+\beta_{n}age_{bins}+\beta_{m}sex$$

With $\beta_{0}$ as constant, $\beta_{1}$ and so on as regression coefficients, $Score_{A}$ as cumulative burden score on pathway A (adapted for pathways occurring in the cohort), and $age_{bins}$ as age in 10-year bins.

As outcomes Y the occurrence of falls respectively haemorrhages in the ADRED cohort was used as described in the methods.

**S2 – Details on the model of falls**

| **Omnibus Test of Model Coefficients** | | | | | | | |  |
| --- | --- | --- | --- | --- | --- | --- | --- | --- |
|  | | | Chi-square | | df | | Sig. |  |
| Step 1 | | Step | 138.342 | | 33 | | <.001 |  |
|  |  | Block | 138.342 | | 33 | | <.001 |  |
|  |  | Model | 138.342 | | 33 | | <.001 |  |
| Step 24 | | Step | -2.646 | | 1 | | .104 |  |
|  |  | Block | 127.921 | | 10 | | <.001 |  |
|  |  | Model | 127.921 | | 10 | | <.001 |  |
|  | | | | | | | |  |
| **Model Summary** | | | | | | | | |
| Step | -2 Log-Likelihood | | | Cox & Snell R Square | | Nagelkerkes R Square | | |
| 1 | 2161.265 | | | .030 | | .075 | | |
| 24 | 2171.686 | | | .028 | | .070 | | |
|  | | | | | | | | |

| **Variables in the Equation** | | | | | | | | | |
| --- | --- | --- | --- | --- | --- | --- | --- | --- | --- |
|  | | B (Coefficient) | S.E. (Standard Error) | Wald | df | Sig. | Exp(B) (Odds Ratio) | 95% Confidence Interval for Exp(B) | |
|  |  |  |  |  |  |  |  | Lower | Upper |
| Step 1 | age_bins | .448 | .053 | 72.054 | 1 | <.001 | 1.565 | 1.411 | 1.736 |
|  | sex | .183 | .122 | 2.231 | 1 | .135 | 1.201 | .945 | 1.526 |
|  | Score_1A1 | -.100 | .252 | .156 | 1 | .693 | .905 | .552 | 1.484 |
|  | Score_1A2 | .011 | .105 | .011 | 1 | .915 | 1.011 | .823 | 1.243 |
|  | Score_2B6 | -.168 | .203 | .683 | 1 | .409 | .846 | .568 | 1.259 |
|  | Score_2C19 | .013 | .088 | .021 | 1 | .885 | 1.013 | .852 | 1.205 |
|  | Score_2C8 | -.261 | .104 | 6.332 | 1 | .012 | .770 | .629 | .944 |
|  | Score_2C9 | -.046 | .105 | .189 | 1 | .663 | .955 | .777 | 1.174 |
|  | Score_2D6 | .134 | .060 | 4.947 | 1 | .026 | 1.143 | 1.016 | 1.286 |
|  | Score_2E1 | -.095 | .119 | .636 | 1 | .425 | .910 | .721 | 1.148 |
|  | Score_3A | .098 | .098 | 1.013 | 1 | .314 | 1.103 | .911 | 1.336 |
|  | Score_BCRP | -.009 | .066 | .018 | 1 | .892 | .991 | .871 | 1.127 |
|  | Score_MRP2 | -.106 | .210 | .254 | 1 | .614 | .900 | .596 | 1.357 |
|  | Score_MRP4 | -.052 | .251 | .042 | 1 | .837 | .950 | .581 | 1.554 |
|  | Score_NET | -.284 | .325 | .764 | 1 | .382 | .753 | .398 | 1.423 |
|  | Score_OAT1 | .162 | .245 | .442 | 1 | .506 | 1.176 | .728 | 1.900 |
|  | Score_OAT2 | -.306 | .264 | 1.351 | 1 | .245 | .736 | .439 | 1.234 |
|  | Score_OAT3 | -.062 | .126 | .244 | 1 | .621 | .940 | .734 | 1.203 |
|  | Score_OAT4 | .171 | .171 | .991 | 1 | .320 | 1.186 | .848 | 1.660 |
|  | Score_OATP1A2 | -.232 | .242 | .917 | 1 | .338 | .793 | .494 | 1.274 |
|  | Score_OATP1B1 | -.154 | .112 | 1.918 | 1 | .166 | .857 | .689 | 1.066 |
|  | Score_OCT1 | -.655 | .454 | 2.080 | 1 | .149 | .520 | .213 | 1.265 |
|  | Score_OCT2 | .327 | .140 | 5.452 | 1 | .020 | 1.387 | 1.054 | 1.826 |
|  | Score_OCT3 | .577 | .468 | 1.522 | 1 | .217 | 1.781 | .712 | 4.455 |
|  | Score_Pgp | -.038 | .077 | .247 | 1 | .619 | .963 | .829 | 1.118 |
|  | Score_SERT | .173 | .370 | .218 | 1 | .640 | 1.189 | .575 | 2.457 |
|  | Score_SGLT2 | .224 | .170 | 1.737 | 1 | .188 | 1.251 | .897 | 1.745 |
|  | Score_UGT2B7 | .059 | .145 | .168 | 1 | .682 | 1.061 | .799 | 1.410 |
|  | Score_VKOR | -.011 | .109 | .011 | 1 | .916 | .989 | .799 | 1.224 |
|  | Score_carboxylesterase1 | .875 | .499 | 3.076 | 1 | .079 | 2.400 | .902 | 6.382 |
|  | Score_carboxylesterase2 | -.800 | .566 | 1.995 | 1 | .158 | .449 | .148 | 1.363 |
|  | Score_dipeptidylpeptidase4 | -.025 | .083 | .091 | 1 | .763 | .975 | .830 | 1.147 |
|  | Score_purinenucleosidephosphorylase | -.153 | .185 | .684 | 1 | .408 | .858 | .597 | 1.233 |
|  | constant | -5.387 | .376 | 205.381 | 1 | <.001 | .005 |  |  |
| Step 24 | age_bins | .455 | .050 | 81.333 | 1 | <.001 | 1.576 | 1.428 | 1.740 |
|  | Score_2B6 | -.196 | .112 | 3.043 | 1 | .081 | .822 | .660 | 1.024 |
|  | Score_2C8 | -.262 | .082 | 10.226 | 1 | .001 | .770 | .656 | .904 |
|  | Score_2D6 | .164 | .052 | 9.994 | 1 | .002 | 1.178 | 1.064 | 1.303 |
|  | Score_OAT2 | -.260 | .142 | 3.354 | 1 | .067 | .771 | .584 | 1.018 |
|  | Score_OATP1B1 | -.157 | .080 | 3.901 | 1 | .048 | .855 | .731 | .999 |
|  | Score_OCT1 | -.366 | .219 | 2.793 | 1 | .095 | .694 | .452 | 1.065 |
|  | Score_OCT2 | .326 | .118 | 7.635 | 1 | .006 | 1.386 | 1.099 | 1.747 |
|  | Score_carboxylesterase1 | .883 | .365 | 5.842 | 1 | .016 | 2.419 | 1.182 | 4.951 |
|  | Score_carboxylesterase2 | -.801 | .379 | 4.457 | 1 | .035 | .449 | .213 | .944 |
|  | constant | -5.156 | .321 | 258.470 | 1 | <.001 | .006 |  |  |

**S3 - Frequently occurring substrates on pathways positively associated with the occurrence of falls, classification into FRID, degree of pathway contribution on substrate metabolism, and inhibitory effect**

| Pathway | Substrate | Degree of Pathway Contribution to Substrate Metabolism | Inhibitory effect of Substrate on Pathway | Frequency in cohort, n (%) |
| --- | --- | --- | --- | --- |
| CYP2D6 | metoclopramide | relevant | moderate | 153 (3.3) |
|  | citalopram^a^ | relevant | moderate | 106 (2.3) |
|  | tamsulosin^a^ | relevant | no impact | 388 (8.5) |
|  | mirtazapine^a^ | relevant | no impact | 258 (5.6) |
|  | risperidone^a^ | main | weak | 112 (2.4) |
|  | metoprolol | main | no clinical relevance | 1083 (23.6) |
|  | carvedilol | main | no impact | 162 (3.5) |
|  | tramadol^a^ | main | no impact | 104 (2.3) |
|  | nebivolol | main | no impact | 98 (2.1) |
|  | escitalopram^a^ | side | weak | 94 (2.1) |
|  | bisoprolol | side | no impact | 902 (19.7) |
|  | simvastatin | side | no impact | 668 (14.6) |
|  | formoterol | side | no impact | 185 (4.0) |
|  | tiotropium bromide | side | no impact | 160 (3.5) |
|  | oxycodone^a^ | side | no impact | 129 (2.8) |
|  | quetiapine^a^ | side | no impact | 128 (2.8) |
| OCT2 | trimethoprim | relevant | moderate | 112 (2.4) |
|  | metformin | relevant | no impact | 566 (12.4) |
|  | tiotropium bromide | relevant | no impact | 160 (3.5) |
|  | carvedilol | side | weak | 162 (3.5) |
| carboxyl-esterase 1 | clopidogrel | relevant | weak | 305 (6.7) |

^a^ FRID according to STOPPFall consensus ^2^

Substrates of significant pathways occurring in the cohort with a frequency of ≥ 2%, the relevance of the pathway on its metabolism and their impact on pathway capacity modulation. Degree of pathway contribution to substrate metabolism classified as main, relevant or side pathway. Inhibitory effect of the substrate on the pathway classified according to mediQ: no clinical relevance for 0/3 inhibitor, weak for 1/3 weak inhibitor, moderate for 2/3 moderate inhibitor, strong for 3/3 strong inhibitor according to mediQ.

| **S4 – Details on the model of haemorrhages**   \| **Omnibus Test of Model Coefficients** \| \| \| \| \| \| --- \| --- \| --- \| --- \| --- \| \|  \| \| Chi-square \| df \| Sig. \| \| Step 1 \| Step \| 401.657 \| 33 \| <.001 \| \| Block \| 401.657 \| 33 \| <.001 \| \| Model \| 401.657 \| 33 \| <.001 \| \| Step 13 \| Step \| -1.993 \| 1 \| .158 \| \| Block \| 395.488 \| 21 \| <.001 \| \| Model \| 395.488 \| 21 \| <.001 \|  \| **Model Summary** \| \| \| \| \| --- \| --- \| --- \| --- \| \| Step \| -2 Log-Likelihood \| Cox & Snell R Square \| Nagelkerkes R Square \| \| 1 \| 5349.354 \| .084 \| .117 \| \| 13 \| 5355.522 \| .083 \| .116 \| \|  \| \| \| \|  \| **Variables in the Equation** \| \| \| \| \| \| \| \| \| \| \| --- \| --- \| --- \| --- \| --- \| --- \| --- \| --- \| --- \| --- \| \|  \| \| B (Coefficient) \| S.E. (Standard Error) \| Wald \| df \| Sig. \| Exp(B) (Odds Ratio) \| 95% Confidence Interval for Exp(B) \| \| \| Lower \| Upper \| \| Step 1 \| age_bins \| .245 \| .026 \| 87.104 \| 1 \| <.001 \| 1.278 \| 1.214 \| 1.346 \| \| sex \| -.365 \| .068 \| 28.605 \| 1 \| <.001 \| .694 \| .607 \| .793 \| \| Score_1A1 \| -.250 \| .127 \| 3.868 \| 1 \| .049 \| .779 \| .607 \| .999 \| \| Score_1A2 \| .048 \| .061 \| .613 \| 1 \| .434 \| 1.049 \| .931 \| 1.182 \| \| Score_2B6 \| -.025 \| .097 \| .065 \| 1 \| .798 \| .976 \| .807 \| 1.179 \| \| Score_2C19 \| .206 \| .051 \| 16.384 \| 1 \| <.001 \| 1.229 \| 1.112 \| 1.359 \| \| Score_2C8 \| .064 \| .055 \| 1.349 \| 1 \| .245 \| 1.066 \| .957 \| 1.188 \| \| Score_2C9 \| -.121 \| .058 \| 4.394 \| 1 \| .036 \| .886 \| .791 \| .992 \| \| Score_2D6 \| -.153 \| .039 \| 15.436 \| 1 \| <.001 \| .858 \| .795 \| .926 \| \| Score_2E1 \| -.166 \| .064 \| 6.766 \| 1 \| .009 \| .847 \| .747 \| .960 \| \| Score_3A \| .076 \| .056 \| 1.839 \| 1 \| .175 \| 1.079 \| .967 \| 1.203 \| \| Score_BCRP \| .001 \| .036 \| .002 \| 1 \| .968 \| 1.001 \| .933 \| 1.075 \| \| Score_MRP2 \| -.029 \| .105 \| .077 \| 1 \| .782 \| .971 \| .791 \| 1.193 \| \| Score_MRP4 \| .357 \| .136 \| 6.943 \| 1 \| .008 \| 1.429 \| 1.096 \| 1.865 \| \| Score_NET \| -.396 \| .188 \| 4.424 \| 1 \| .035 \| .673 \| .465 \| .973 \| \| Score_OAT1 \| -.276 \| .129 \| 4.541 \| 1 \| .033 \| .759 \| .589 \| .978 \| \| Score_OAT2 \| .433 \| .150 \| 8.384 \| 1 \| .004 \| 1.542 \| 1.150 \| 2.068 \| \| Score_OAT3 \| .083 \| .071 \| 1.362 \| 1 \| .243 \| 1.086 \| .945 \| 1.248 \| \| Score_OAT4 \| .032 \| .099 \| .105 \| 1 \| .746 \| 1.032 \| .851 \| 1.253 \| \| Score_OATP1A2 \| -.256 \| .134 \| 3.649 \| 1 \| .056 \| .774 \| .596 \| 1.007 \| \| Score_OATP1B1 \| .154 \| .058 \| 7.040 \| 1 \| .008 \| 1.167 \| 1.041 \| 1.308 \| \| Score_OCT1 \| -.114 \| .214 \| .282 \| 1 \| .595 \| .893 \| .587 \| 1.357 \| \| Score_OCT2 \| .069 \| .082 \| .697 \| 1 \| .404 \| 1.071 \| .912 \| 1.259 \| \| Score_OCT3 \| .236 \| .250 \| .893 \| 1 \| .345 \| 1.266 \| .776 \| 2.065 \| \| Score_Pgp \| -.083 \| .044 \| 3.593 \| 1 \| .058 \| .921 \| .845 \| 1.003 \| \| Score_SERT \| .536 \| .210 \| 6.487 \| 1 \| .011 \| 1.709 \| 1.131 \| 2.580 \| \| Score_SGLT2 \| -.115 \| .104 \| 1.218 \| 1 \| .270 \| .891 \| .727 \| 1.093 \| \| Score_UGT2B7 \| -.180 \| .089 \| 4.089 \| 1 \| .043 \| .835 \| .701 \| .994 \| \| Score_VKOR \| .379 \| .060 \| 39.688 \| 1 \| <.001 \| 1.461 \| 1.299 \| 1.644 \| \| Score_carboxylesterase1 \| .283 \| .235 \| 1.456 \| 1 \| .228 \| 1.327 \| .838 \| 2.103 \| \| Score_carboxylesterase2 \| -.121 \| .269 \| .203 \| 1 \| .652 \| .886 \| .523 \| 1.500 \| \| Score_dipeptidylpeptidase4 \| .047 \| .046 \| 1.038 \| 1 \| .308 \| 1.048 \| .958 \| 1.146 \| \| Score_purinenucleosidephosphorylase \| -.047 \| .097 \| .234 \| 1 \| .628 \| .954 \| .788 \| 1.155 \| \| constant \| -1.891 \| .180 \| 109.941 \| 1 \| <.001 \| .151 \|  \|  \| \| Step 13 \| age_bins \| .248 \| .026 \| 93.708 \| 1 \| <.001 \| 1.282 \| 1.219 \| 1.348 \| \| sex \| -.349 \| .067 \| 26.961 \| 1 \| <.001 \| .705 \| .618 \| .805 \| \| Score_1A1 \| -.285 \| .086 \| 10.961 \| 1 \| <.001 \| .752 \| .635 \| .890 \| \| Score_2C19 \| .212 \| .048 \| 19.300 \| 1 \| <.001 \| 1.236 \| 1.124 \| 1.358 \| \| Score_2C9 \| -.103 \| .048 \| 4.627 \| 1 \| .031 \| .902 \| .821 \| .991 \| \| Score_2D6 \| -.146 \| .037 \| 15.556 \| 1 \| <.001 \| .864 \| .804 \| .929 \| \| Score_2E1 \| -.167 \| .063 \| 7.071 \| 1 \| .008 \| .846 \| .749 \| .957 \| \| Score_3A \| .099 \| .051 \| 3.813 \| 1 \| .051 \| 1.104 \| 1.000 \| 1.219 \| \| Score_MRP4 \| .383 \| .115 \| 11.105 \| 1 \| <.001 \| 1.467 \| 1.171 \| 1.839 \| \| Score_NET \| -.270 \| .146 \| 3.417 \| 1 \| .065 \| .764 \| .574 \| 1.016 \| \| Score_OAT1 \| -.337 \| .101 \| 11.234 \| 1 \| <.001 \| .714 \| .586 \| .869 \| \| Score_OAT2 \| .408 \| .140 \| 8.490 \| 1 \| .004 \| 1.504 \| 1.143 \| 1.979 \| \| Score_OAT3 \| .090 \| .051 \| 3.090 \| 1 \| .079 \| 1.094 \| .990 \| 1.208 \| \| Score_OATP1A2 \| -.206 \| .101 \| 4.174 \| 1 \| .041 \| .813 \| .667 \| .992 \| \| Score_OATP1B1 \| .166 \| .046 \| 12.847 \| 1 \| <.001 \| 1.180 \| 1.078 \| 1.292 \| \| Score_OCT2 \| .115 \| .070 \| 2.733 \| 1 \| .098 \| 1.122 \| .979 \| 1.286 \| \| Score_Pgp \| -.082 \| .035 \| 5.523 \| 1 \| .019 \| .921 \| .860 \| .986 \| \| Score_SERT \| .420 \| .176 \| 5.695 \| 1 \| .017 \| 1.522 \| 1.078 \| 2.148 \| \| Score_UGT2B7 \| -.189 \| .087 \| 4.760 \| 1 \| .029 \| .828 \| .699 \| .981 \| \| Score_VKOR \| .378 \| .060 \| 39.912 \| 1 \| <.001 \| 1.460 \| 1.298 \| 1.641 \| \| Score_carboxylesterase1 \| .274 \| .101 \| 7.398 \| 1 \| .007 \| 1.315 \| 1.079 \| 1.601 \| \| constant \| -1.914 \| .178 \| 116.080 \| 1 \| <.001 \| .147 \|  \|  \| \|  \| \| \| \| \| \| \| \| \| \| |
| --- | --- | --- | --- | --- | --- | --- | --- | --- | --- | --- | --- | --- | --- | --- | --- | --- | --- | --- | --- | --- | --- | --- | --- | --- | --- | --- | --- | --- | --- | --- | --- | --- | --- | --- | --- | --- | --- | --- | --- | --- | --- | --- | --- | --- | --- | --- | --- | --- | --- | --- | --- | --- | --- | --- | --- | --- | --- | --- | --- | --- | --- | --- | --- | --- | --- | --- | --- | --- | --- | --- | --- | --- | --- | --- | --- | --- | --- | --- | --- | --- | --- | --- | --- | --- | --- | --- | --- | --- | --- | --- | --- | --- | --- | --- | --- | --- | --- | --- | --- | --- | --- | --- | --- | --- | --- | --- | --- | --- | --- | --- | --- | --- | --- | --- | --- | --- | --- | --- | --- | --- | --- | --- | --- | --- | --- | --- | --- | --- | --- | --- | --- | --- | --- | --- | --- | --- | --- | --- | --- | --- | --- | --- | --- | --- | --- | --- | --- | --- | --- | --- | --- | --- | --- | --- | --- | --- | --- | --- | --- | --- | --- | --- | --- | --- | --- | --- | --- | --- | --- | --- | --- | --- | --- | --- | --- | --- | --- | --- | --- | --- | --- | --- | --- | --- | --- | --- | --- | --- | --- | --- | --- | --- | --- | --- | --- | --- | --- | --- | --- | --- | --- | --- | --- | --- | --- | --- | --- | --- | --- | --- | --- | --- | --- | --- | --- | --- | --- | --- | --- | --- | --- | --- | --- | --- | --- | --- | --- | --- | --- | --- | --- | --- | --- | --- | --- | --- | --- | --- | --- | --- | --- | --- | --- | --- | --- | --- | --- | --- | --- | --- | --- | --- | --- | --- | --- | --- | --- | --- | --- | --- | --- | --- | --- | --- | --- | --- | --- | --- | --- | --- | --- | --- | --- | --- | --- | --- | --- | --- | --- | --- | --- | --- | --- | --- | --- | --- | --- | --- | --- | --- | --- | --- | --- | --- | --- | --- | --- | --- | --- | --- | --- | --- | --- | --- | --- | --- | --- | --- | --- | --- | --- | --- | --- | --- | --- | --- | --- | --- | --- | --- | --- | --- | --- | --- | --- | --- | --- | --- | --- | --- | --- | --- | --- | --- | --- | --- | --- | --- | --- | --- | --- | --- | --- | --- | --- | --- | --- | --- | --- | --- | --- | --- | --- | --- | --- | --- | --- | --- | --- | --- | --- | --- | --- | --- | --- | --- | --- | --- | --- | --- | --- | --- | --- | --- | --- | --- | --- | --- | --- | --- | --- | --- | --- | --- | --- | --- | --- | --- | --- | --- | --- | --- | --- | --- | --- | --- | --- | --- | --- | --- | --- | --- | --- | --- | --- | --- | --- | --- | --- | --- | --- | --- | --- | --- | --- | --- | --- | --- | --- | --- | --- | --- | --- | --- | --- | --- | --- | --- | --- | --- | --- | --- | --- | --- | --- | --- | --- | --- | --- | --- | --- | --- | --- | --- | --- | --- | --- | --- | --- | --- | --- | --- | --- | --- | --- | --- | --- | --- | --- | --- | --- | --- | --- | --- | --- | --- | --- | --- | --- | --- | --- | --- | --- | --- | --- | --- | --- | --- | --- | --- | --- | --- | --- | --- | --- | --- | --- | --- | --- | --- | --- | --- | --- | --- | --- | --- | --- | --- | --- | --- | --- | --- | --- | --- | --- | --- | --- | --- | --- | --- | --- | --- | --- | --- | --- | --- | --- | --- | --- | --- | --- | --- | --- | --- | --- | --- | --- | --- | --- | --- | --- | --- | --- | --- | --- | --- | --- | --- | --- | --- | --- | --- | --- | --- | --- | --- | --- | --- | --- | --- | --- | --- | --- | --- | --- | --- | --- | --- | --- | --- | --- | --- | --- | --- | --- | --- | --- | --- | --- | --- | --- | --- | --- | --- | --- | --- | --- | --- | --- | --- | --- | --- | --- | --- | --- | --- | --- | --- | --- | --- | --- | --- | --- | --- |

**S5 - Frequently occurring substrates on pathways positively associated with the occurrence of haemorrhages, degree of pathway contribution on substrate metabolism, and inhibitory effect**

| Pathway | Substrate | Degree of Pathway Contribution to Substrate Metabolism | Inhibitory effect of Substrate on Pathway | Relative frequency in cohort (%) |
| --- | --- | --- | --- | --- |
| CYP2C19 | pantoprazole^b^ | relevant | weak | 1672 (36.5) |
|  | apixaban^a^ | side | weak | 547 (11.9) |
|  | clopidogrel^a^ | relevant | weak | 305 (6.7) |
|  | citalopram^a^ | relevant | weak | 106 (2.3) |
|  | escitalopram^a^ | relevant | weak | 94 (2.1) |
|  | omeprazole^b^ | main | moderate | 157 (3.4) |
|  | tilidine | relevant | no impact | 331 (7.2) |
|  | ibuprofen^a^ | side | no impact | 275 (6.0) |
|  | formoterol | side | no impact | 185 (4.0) |
|  | carvedilol^b^ | side | no impact | 162 (3.5) |
| CYP3A | bisoprolol^b^ | relevant | no impact | 902 (19.7) |
|  | amlodipine^b^ | relevant | weak | 753 (16.4) |
|  | apixaban^a^ | relevant | no impact | 547 (11.9) |
|  | tamsulosin | relevant | no impact | 388 (8.5) |
|  | tilidine | relevant | weak | 331 (7.2) |
|  | phenprocoumon^a^ | relevant | no impact | 331 (7.2) |
|  | clopidogrel^a^ | relevant | no impact | 305 (6.7) |
|  | rivaroxaban^a^ | relevant | no impact | 259 (5.7) |
|  | mirtazapine | relevant | no impact | 258 (5.6) |
|  | digitoxin^b^ | relevant | no impact | 165 (3.6) |
|  | omeprazole^b^ | relevant | weak | 157 (3.4) |
|  | fentanyl | relevant | weak | 116 (2.5) |
|  | risperidone | relevant | no clinical relevance | 112 (2.4) |
|  | citalopram^a^ | relevant | no clinical relevance | 106 (2.3) |
|  | zopiclone | relevant | no impact | 102 (2.2) |
|  | atorvastatin^b^ | main | weak | 847 (18.5) |
|  | simvastatin^b^ | main | no impact | 668 (14.6) |
|  | prednisolone | main | weak inducing | 410 (9.0) |
|  | lercanidipine^b^ | main | no impact | 251 (5.5) |
|  | budesonide | main | weak inducing | 137 (3.0) |
|  | oxycodone | main | no impact | 129 (2.8) |
|  | quetiapine | main | no impact | 128 (2.8) |
|  | eplerenone^b^ | main | no impact | 106 (2.3) |
|  | nitrendipine^b^ | main | no impact | 99 (2.2) |
|  | pantoprazole^b^ | side | no impact | 1672 (36.5) |
|  | sitagliptin | side | weak | 316 (6.9) |
|  | edoxaban^a^ | side | no impact | 275 (6.0) |
|  | ibuprofen^a^ | side | no impact | 275 (6.0) |
|  | carvedilol^b^ | side | no impact | 162 (3.5) |
|  | tiotropium bromide | side | no impact | 160 (3.5) |
|  | beclomethasone | side | no impact | 115 (2.5) |
|  | tramadol | side | no impact | 104 (2.3) |
|  | escitalopram^a^ | side | no impact | 94 (2.0) |
| MRP4 | acetylsalicylic acid^a^ | side | weak | 1375 (30.0) |
|  | furosemide^b^ | side | weak | 157 (3.4) |
| OAT2 | acetylsalicylic acid^a^ | side | weak | 1375 (30.0) |
|  | allopurinol | side | no impact | 610 (13.3) |
| OATP1B1 | torasemide^b^ | relevant | no impact | 1448 (31.6) |
|  | simvastatin^b^ | relevant | no impact | 668 (14.6) |
|  | ezetimibe^b^ | relevant | weak | 198 (4.3) |
|  | atorvastatin^b^ | main | no impact | 847 (18.5) |
|  | rosuvastatin^b^ | main | no impact | 139 (3.0) |
|  | valsartan^b^ | side | moderate | 296 (6.5) |
| carboxyl-esterase 1 | clopidogrel^a^ | relevant | weak | 305 (6.7) |

^a^ drug with hemorrhage as known side effect ^b^ drug known to be prescribed together with anticoagulant according to medical guidelines^3^

Substrates of significant pathways occurring in the cohort with a frequency of ≥ 2%, the relevance of the pathway on its metabolism and their impact on pathway capacity modulation. Degree of pathway contribution to substrate metabolism classified as main, relevant or side pathway. Inhibitory effect of the substrate on the pathway classified according to mediQ: no clinical relevance for 0/3 inhibitor, weak for 1/3 weak inhibitor, moderate for 2/3 moderate inhibitor, strong for 3/3 strong inhibitor according to mediQ.

**References**

1. Zürich U. Logistische Regressionsanalyse. Accessed 20.06., 2025. <https://www.methodenberatung.uzh.ch/de/datenanalyse_spss/zusammenhaenge/lreg.html>

2. Seppala LJ, Petrovic M, Ryg J, et al. STOPPFall (Screening Tool of Older Persons Prescriptions in older adults with high fall risk): a Delphi study by the EuGMS Task and Finish Group on Fall-Risk-Increasing Drugs. *Age and Ageing*. 2020;50(4):1189-1199. doi:10.1093/ageing/afaa249

3. Vrints C, Andreotti F, Koskinas KC, et al. 2024 ESC Guidelines for the management of chronic coronary syndromes: Developed by the task force for the management of chronic coronary syndromes of the European Society of Cardiology (ESC) Endorsed by the European Association for Cardio-Thoracic Surgery (EACTS). *European Heart Journal*. 2024;45(36):3415-3537. doi:10.1093/eurheartj/ehae177
